# *trans*-Diaqua­bis­[5-(pyridine-3-carboxamido)­tetra­zolido-κ^2^ *O*,*N* ^1^]zinc dihydrate

**DOI:** 10.1107/S1600536812014997

**Published:** 2012-04-21

**Authors:** Fang Li, Xiang-Ping Ou, Chang-Cang Huang

**Affiliations:** a State Key Laboratory Breeding Base of Photocatalysis, College of Chemistry & Chemical Engineering, Fuzhou University, Fuzhou, Fujian 350108, People’s Republic of China

## Abstract

The title compound, [Zn(C_7_H_5_N_6_O)_2_(H_2_O)_2_]·2H_2_O, consists of one Zn^II^ ion located on the crystallographic inversion centre, two 5-(pyridine-3-carboxamido)­tetra­zolide ligands, two coordinated water mol­ecules and two free water mol­ecules. The Zn^II^ ion adopts a slightly distorted octa­hedral coordination geometry formed by the *N*,*O*-chelating ligands and two O water atoms. The pyridine N atoms are not coordinated. In the crystal, complex mol­ecules are connected by N—H⋯O, O—H⋯N and O—H⋯O hydrogen bonds, forming a three-dimensional network.

## Related literature

For pharmaceutical applications of amide derivatives, see: Foster *et al.* (1999 ▶); Rauko *et al.* (2001 ▶); Rowland *et al.* (2001 ▶, 2002 ▶). For our recent work on the design and synthesis of amide complexes, see: Wang *et al.* (2010 ▶). For the use of nicotinoyl­amino in building novel complexes, see: Aakeröy *et al.* (2001 ▶); Li *et al.* (2008 ▶); Moncol *et al.* (2007 ▶); Kumar *et al.* (2005 ▶). For Zn—N and Zn—O bond lengths in related structures, see: Armstrong *et al.* (2003 ▶); Liu *et al.* (2009 ▶).
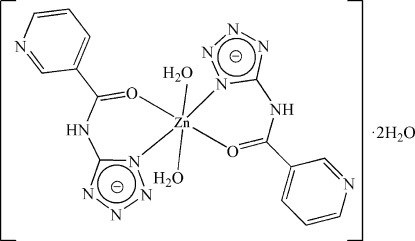


## Experimental

### 

#### Crystal data

[Zn(C_7_H_5_N_6_O)_2_(H_2_O)_2_]·2H_2_O
*M* *_r_* = 515.79
Monoclinic, 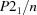

*a* = 7.2576 (15) Å
*b* = 12.008 (2) Å
*c* = 11.917 (2) Å
β = 97.76 (3)°
*V* = 1029.1 (3) Å^3^
*Z* = 2
Mo *K*α radiation
μ = 1.26 mm^−1^
*T* = 293 K
0.22 × 0.15 × 0.1 mm

#### Data collection

Rigaku Saturn 724 CCD area-detector diffractometer
Absorption correction: multi-scan (*CrystalClear*; Rigaku, 2002 ▶) *T*_min_ = 0.819, *T*_max_ = 1.000
8464 measured reflections
2374 independent reflections
2188 reflections with *I* > 2σ(*I*)
*R*_int_ = 0.043

#### Refinement

*R*[*F* ^2^ > 2σ(*F* ^2^)] = 0.045
*wR*(*F* ^2^) = 0.100
*S* = 1.19
2374 reflections
165 parameters
6 restraints
H atoms treated by a mixture of independent and constrained refinement
Δρ_max_ = 0.24 e Å^−3^
Δρ_min_ = −0.33 e Å^−3^

### 

Data collection: *CrystalClear* (Rigaku, 2002 ▶); cell refinement: *CrystalClear*; data reduction: *CrystalClear*; program(s) used to solve structure: *SHELXTL* (Sheldrick, 2008 ▶); program(s) used to refine structure: *SHELXTL*; molecular graphics: *SHELXTL*; software used to prepare material for publication: *SHELXTL*.

## Supplementary Material

Crystal structure: contains datablock(s) I, global. DOI: 10.1107/S1600536812014997/hg5200sup1.cif

Structure factors: contains datablock(s) I. DOI: 10.1107/S1600536812014997/hg5200Isup2.hkl

Additional supplementary materials:  crystallographic information; 3D view; checkCIF report

## Figures and Tables

**Table 1:** Selected bond lengths (Å)

Zn1—N2	2.058 (2)
Zn1—O2	2.131 (2)
Zn1—O1	2.1470 (17)

**Table 2:** Hydrogen-bond geometry (Å, °)

*D*—H⋯*A*	*D*—H	H⋯*A*	*D*⋯*A*	*D*—H⋯*A*
N1—H6⋯O3	0.86	2.04	2.829 (3)	153
O2—H2*A*⋯N5^i^	0.84 (1)	1.97 (1)	2.795 (3)	165 (3)
O2—H2*B*⋯N6^ii^	0.84 (1)	1.89 (1)	2.727 (3)	178 (3)
O3—H3*A*⋯O2^i^	0.84 (1)	2.08 (2)	2.843 (3)	152 (4)
O3—H3*B*⋯N4^iii^	0.84 (1)	2.09 (1)	2.907 (3)	164 (4)

Symmetry codes: (i) 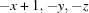; (ii) 
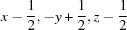
; (iii) 
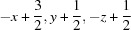
.

**Fig. 1.**
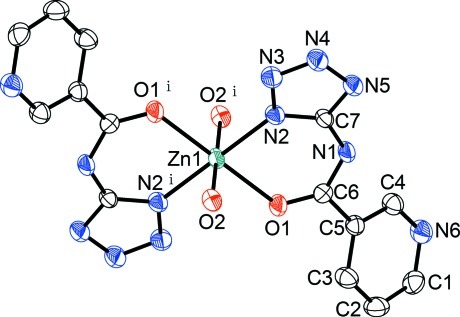
View of the coordination environment of Zn2+ in title compound at 50%. H atoms have been omitted for clarity. [Symmetry codes: (i) -x, -y, -z.]

**Fig. 2.**
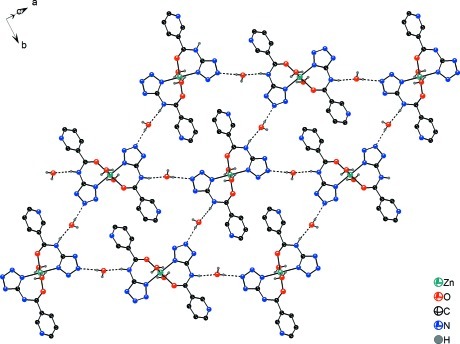
A view of the two-dimensional structure formed via hydrogen bonds.

## supplementary crystallographic information

### Comment

In recent years, many metal compounds derived from amides, have been prepared and characterized, and have been found to possess a wide variety of pharmic applications (Foster *et al.*, 1999; Rauko *et al.*, 2001; Rowland *et al.*, 2001; Rowland *et al.*, 2002). Amides were used to construct extended frameworks sustained both by hydrogen bonds and coordination bonds owing to the inherent coordination and hydrogenbonding donor/acceptor functionalities (Aakeröy *et al.*, 2001; Li *et al.*, 2008; Moncol *et al.*, 2007; Kumar *et al.*, 2005). In this paper, we report the crystal structure of the zinc-amide complex.

The asymmetric unit of complex, (I), contains one Zn^II^ ion located on an inversion centre, one independent *N*-(tetrazol-5-yl)-nicotinamide ligand, one coordination water molecule and one free water molecule. The central Zn^II^ ion adopts a slightly distorted octahedral coordination geometry by two ligands and two coordination water molecules. The equatorial plane is formed by two tetrazole N atoms and two O atoms in bis-N, O-chelating coordination from two *N*-(tetrazol-5-yl)-nicotinamide ligands, while the axial positions are occupied by two O atoms from two coordination water molecules. The Zn–N bond length is 2.058 (2) Å. The bond lengths of Zn–O are in the range 2.131 (2)–2.147 (1) Å. All the Zn–N or Zn–O bond lengths are comparable to those reported previously for zinc compounds (Armstrong *et al.*, 2003; Liu *et al.*, 2009). The dihedral angle between tetrazole and pyridine groups is 45.988 (1)°.

In the crystal, the complex molecules are connected to a two dimensional layer by intermolecular N–H···O_free_ (O atoms from free water molecules) hydrogen bonds (Fig. 2, Table 2). The layered strcture form a three-dimensional network *via* O_free_–H···O_coord_ (O atoms from coordination water molecules), O_free_–H···N and O_coord_–H···N hydrogen bonds.

### Experimental

The title compound was synthesized by reacting the ligand (*N*-(1*H*-tetrazol-5-yl)-nicotinamide) (0.019 g,0.01 mmol) with Zn(CH_3_COO)_2_^.^2H_2_O (0.011 g, 0.05 mmol) in 5.0 ml of dimethyl sulfoxide followed by the addition of 4 ml of ethanol. The muddy solution obtained was stirred at room temperature for three hours, filtered and set aside to slowly crystallize at room temperature. The block-like crystals were obtained after about three weeks.

### Refinement

Four reflections, -2 1 1, -5 5 2, -5 2 3, -1 0 1, shaded by beamstop were omitted. All H atoms bonded to C and N atoms were refined in idealized positions using the riding-model approximation, with C–H = 0.93 Å, *U*_iso_(H) = 1.2 *U*_eq_(C) and N–H = 0.93 Å, *U*_iso_(H) = 1.2 *U*_eq_(N). In water molecule the O–H distances were restrained to 0.84 (5) Å, and the distance H···H to 1.32 (2) Å, with *U*_iso_(H) = 1.5 *U*_eq_(O).

### Crystal data

**Table:**

[Zn(C_7_H_5_N_6_O)_2_(H_2_O)_2_]·2H_2_O	*F*(000) = 528
*M**_r_* = 515.79	*D*_x_ = 1.665 Mg m^−^^3^
Monoclinic, *P*2_1_/*n*	Mo *K*α radiation, λ = 0.71073 Å
Hall symbol: -P 2yn	Cell parameters from 3552 reflections
*a* = 7.2576 (15) Å	θ = 3.1–27.5°
*b* = 12.008 (2) Å	µ = 1.26 mm^−^^1^
*c* = 11.917 (2) Å	*T* = 293 K
β = 97.76 (3)°	Prism, colorless
*V* = 1029.1 (3) Å^3^	0.22 × 0.15 × 0.1 mm
*Z* = 2	

### Data collection

**Table:**

Rigaku Saturn 724 CCD area-detector diffractometer	2374 independent reflections
Radiation source: fine-focus sealed tube	2188 reflections with *I* > 2σ(*I*)
Graphite monochromator	*R*_int_ = 0.043
scintillation counter scans	θ_max_ = 27.6°, θ_min_ = 3.3°
Absorption correction: multi-scan (*CrystalClear*; Rigaku, 2002)	*h* = −9→7
*T*_min_ = 0.819, *T*_max_ = 1.000	*k* = −15→14
8464 measured reflections	*l* = −15→15

### Refinement

**Table:**

Refinement on *F*^2^	Primary atom site location: structure-invariant direct methods
Least-squares matrix: full	Secondary atom site location: difference Fourier map
*R*[*F*^2^ > 2σ(*F*^2^)] = 0.045	Hydrogen site location: inferred from neighbouring sites
*wR*(*F*^2^) = 0.100	H atoms treated by a mixture of independent and constrained refinement
*S* = 1.19	*w* = 1/[σ^2^(*F*_o_^2^) + (0.0354*P*)^2^ + 0.6596*P*] where *P* = (*F*_o_^2^ + 2*F*_c_^2^)/3
2374 reflections	(Δ/σ)_max_ = 0.042
165 parameters	Δρ_max_ = 0.24 e Å^−^^3^
6 restraints	Δρ_min_ = −0.33 e Å^−^^3^

### Special details

**Table:**

Geometry. All e.s.d.'s (except the e.s.d. in the dihedral angle between two l.s. planes) are estimated using the full covariance matrix. The cell e.s.d.'s are taken into account individually in the estimation of e.s.d.'s in distances, angles and torsion angles; correlations between e.s.d.'s in cell parameters are only used when they are defined by crystal symmetry. An approximate (isotropic) treatment of cell e.s.d.'s is used for estimating e.s.d.'s involving l.s. planes.
Refinement. Refinement of *F*^2^ against ALL reflections. The weighted *R*-factor *wR* and goodness of fit *S* are based on *F*^2^, conventional *R*-factors *R* are based on *F*, with *F* set to zero for negative *F*^2^. The threshold expression of *F*^2^ > σ(*F*^2^) is used only for calculating *R*-factors(gt) *etc*. and is not relevant to the choice of reflections for refinement. *R*-factors based on *F*^2^ are statistically about twice as large as those based on *F*, and *R*- factors based on ALL data will be even larger.

### Fractional atomic coordinates and isotropic or equivalent isotropic displacement parameters (Å^2^)

**Table:**

	*x*	*y*	*z*	*U*_iso_*/*U*_eq_	
Zn1	0.0000	0.0000	0.0000	0.02817 (14)	
O1	0.1128 (2)	0.16405 (14)	−0.01319 (16)	0.0348 (4)	
C5	0.2633 (3)	0.3319 (2)	0.0481 (2)	0.0274 (5)	
C7	0.3943 (3)	0.0364 (2)	0.1181 (2)	0.0259 (5)	
C6	0.2446 (3)	0.2086 (2)	0.0460 (2)	0.0281 (5)	
N4	0.5071 (3)	−0.12357 (19)	0.1550 (2)	0.0364 (5)	
N5	0.5487 (3)	−0.01344 (18)	0.1645 (2)	0.0342 (5)	
N6	0.3527 (3)	0.49961 (18)	0.1502 (2)	0.0370 (5)	
C4	0.3380 (4)	0.3890 (2)	0.1451 (2)	0.0333 (6)	
H12	0.3799	0.3480	0.2097	0.040*	
C3	0.1991 (4)	0.3936 (2)	−0.0474 (2)	0.0349 (6)	
H13	0.1448	0.3584	−0.1132	0.042*	
C1	0.2944 (4)	0.5576 (2)	0.0566 (2)	0.0377 (6)	
H15	0.3064	0.6347	0.0586	0.045*	
C2	0.2168 (4)	0.5077 (2)	−0.0436 (3)	0.0415 (7)	
H16	0.1772	0.5506	−0.1072	0.050*	
N1	0.3798 (3)	0.15158 (17)	0.11143 (18)	0.0309 (5)	
H6	0.4637	0.1899	0.1523	0.037*	
N3	0.3383 (3)	−0.13789 (18)	0.1054 (2)	0.0344 (5)	
N2	0.2617 (3)	−0.03656 (18)	0.08075 (18)	0.0279 (4)	
O2	0.0833 (3)	−0.05113 (17)	−0.15682 (16)	0.0340 (4)	
H2A	0.186 (2)	−0.025 (3)	−0.169 (3)	0.066 (12)*	
H2B	0.011 (3)	−0.037 (3)	−0.2160 (16)	0.061 (11)*	
O3	0.7255 (3)	0.25284 (18)	0.1891 (2)	0.0517 (6)	
H3A	0.801 (4)	0.208 (3)	0.166 (3)	0.078*	
H3B	0.787 (4)	0.287 (3)	0.243 (2)	0.078*	

### Atomic displacement parameters (Å^2^)

**Table:**

	*U*^11^	*U*^22^	*U*^33^	*U*^12^	*U*^13^	*U*^23^
Zn1	0.0224 (2)	0.0244 (2)	0.0352 (2)	−0.00194 (15)	−0.00520 (16)	−0.00004 (16)
O1	0.0314 (10)	0.0247 (9)	0.0444 (11)	−0.0060 (7)	−0.0087 (8)	0.0033 (8)
C5	0.0270 (12)	0.0251 (12)	0.0292 (12)	−0.0025 (9)	0.0006 (10)	−0.0005 (10)
C7	0.0250 (12)	0.0264 (11)	0.0248 (11)	−0.0005 (9)	−0.0015 (9)	0.0007 (9)
C6	0.0298 (13)	0.0257 (12)	0.0284 (12)	−0.0027 (10)	0.0020 (10)	0.0018 (10)
N4	0.0299 (12)	0.0346 (12)	0.0426 (13)	0.0043 (9)	−0.0025 (10)	0.0052 (10)
N5	0.0297 (12)	0.0320 (12)	0.0382 (13)	−0.0006 (9)	−0.0055 (9)	0.0048 (9)
N6	0.0414 (14)	0.0302 (12)	0.0374 (12)	−0.0022 (10)	−0.0019 (10)	−0.0053 (9)
C4	0.0377 (15)	0.0295 (13)	0.0313 (13)	−0.0005 (11)	−0.0007 (11)	−0.0019 (10)
C3	0.0400 (15)	0.0312 (13)	0.0312 (13)	−0.0004 (11)	−0.0030 (11)	−0.0018 (10)
C1	0.0411 (16)	0.0248 (13)	0.0466 (17)	−0.0026 (11)	0.0042 (13)	−0.0005 (11)
C2	0.0529 (18)	0.0323 (15)	0.0372 (15)	0.0014 (12)	−0.0014 (13)	0.0083 (11)
N1	0.0290 (11)	0.0254 (10)	0.0348 (11)	−0.0046 (9)	−0.0083 (9)	−0.0016 (9)
N3	0.0317 (12)	0.0257 (11)	0.0437 (13)	0.0020 (9)	−0.0026 (10)	0.0036 (9)
N2	0.0253 (11)	0.0243 (10)	0.0325 (11)	−0.0001 (8)	−0.0023 (9)	0.0008 (8)
O2	0.0280 (10)	0.0396 (11)	0.0332 (10)	−0.0005 (8)	−0.0005 (8)	−0.0006 (8)
O3	0.0387 (12)	0.0347 (12)	0.0765 (17)	−0.0038 (9)	−0.0109 (11)	−0.0120 (10)

### Geometric parameters (Å, º)

**Table:**

Zn1—N2	2.058 (2)	N4—N5	1.358 (3)
Zn1—N2^i^	2.058 (2)	N6—C4	1.333 (3)
Zn1—O2^i^	2.131 (2)	N6—C1	1.334 (4)
Zn1—O2	2.131 (2)	C4—H12	0.9300
Zn1—O1	2.1470 (17)	C3—C2	1.376 (4)
Zn1—O1^i^	2.1470 (17)	C3—H13	0.9300
O1—C6	1.232 (3)	C1—C2	1.386 (4)
C5—C3	1.385 (4)	C1—H15	0.9300
C5—C4	1.389 (3)	C2—H16	0.9300
C5—C6	1.487 (3)	N1—H6	0.8600
C7—N5	1.323 (3)	N3—N2	1.353 (3)
C7—N2	1.332 (3)	O2—H2A	0.840 (5)
C7—N1	1.389 (3)	O2—H2B	0.839 (5)
C6—N1	1.354 (3)	O3—H3A	0.837 (5)
N4—N3	1.298 (3)	O3—H3B	0.838 (5)
			
N2—Zn1—N2^i^	180.00 (16)	C7—N5—N4	103.8 (2)
N2—Zn1—O2^i^	90.26 (8)	C4—N6—C1	117.9 (2)
N2^i^—Zn1—O2^i^	89.74 (8)	N6—C4—C5	123.3 (2)
N2—Zn1—O2	89.74 (8)	N6—C4—H12	118.3
N2^i^—Zn1—O2	90.26 (8)	C5—C4—H12	118.3
O2^i^—Zn1—O2	180.0	C2—C3—C5	119.0 (3)
N2—Zn1—O1	83.86 (8)	C2—C3—H13	120.5
N2^i^—Zn1—O1	96.14 (8)	C5—C3—H13	120.5
O2^i^—Zn1—O1	87.47 (8)	N6—C1—C2	122.7 (2)
O2—Zn1—O1	92.53 (8)	N6—C1—H15	118.6
N2—Zn1—O1^i^	96.14 (8)	C2—C1—H15	118.6
N2^i^—Zn1—O1^i^	83.86 (8)	C3—C2—C1	119.0 (3)
O2^i^—Zn1—O1^i^	92.53 (8)	C3—C2—H16	120.5
O2—Zn1—O1^i^	87.47 (8)	C1—C2—H16	120.5
O1—Zn1—O1^i^	180.00 (11)	C6—N1—C7	125.5 (2)
C6—O1—Zn1	129.12 (16)	C6—N1—H6	117.3
C3—C5—C4	118.1 (2)	C7—N1—H6	117.3
C3—C5—C6	120.0 (2)	N4—N3—N2	108.3 (2)
C4—C5—C6	122.0 (2)	C7—N2—N3	105.2 (2)
N5—C7—N2	112.0 (2)	C7—N2—Zn1	126.55 (18)
N5—C7—N1	121.8 (2)	N3—N2—Zn1	128.27 (16)
N2—C7—N1	126.1 (2)	Zn1—O2—H2A	114 (2)
O1—C6—N1	123.8 (2)	Zn1—O2—H2B	117 (2)
O1—C6—C5	120.3 (2)	H2A—O2—H2B	104.1 (12)
N1—C6—C5	115.9 (2)	H3A—O3—H3B	104.7 (13)
N3—N4—N5	110.7 (2)		

Symmetry code: (i) −*x*, −*y*, −*z*.

### Hydrogen-bond geometry (Å, º)

**Table:**

*D*—H···*A*	*D*—H	H···*A*	*D*···*A*	*D*—H···*A*
N1—H6···O3	0.86	2.04	2.829 (3)	153
O2—H2*A*···N5^ii^	0.84 (1)	1.97 (1)	2.795 (3)	165 (3)
O2—H2*B*···N6^iii^	0.84 (1)	1.89 (1)	2.727 (3)	178 (3)
O3—H3*A*···O2^ii^	0.84 (1)	2.08 (2)	2.843 (3)	152 (4)
O3—H3*B*···N4^iv^	0.84 (1)	2.09 (1)	2.907 (3)	164 (4)

Symmetry codes: (ii) −*x*+1, −*y*, −*z*; (iii) *x*−1/2, −*y*+1/2, *z*−1/2; (iv) −*x*+3/2, *y*+1/2, −*z*+1/2.
